# Dataset of ultrasonic frequency – domain signals and machine – learning outputs for parameterising lithium – ion battery electrodes’ coating and calendering processes

**DOI:** 10.1016/j.dib.2025.112433

**Published:** 2026-01-03

**Authors:** Erdogan Guk, Mona Faraji Niri, Hamidreza Farhadi Tolie, James Marco

**Affiliations:** a Warwick Manufacturing Group (WMG), University of Warwick, Coventry CV4 7AL, United Kingdom; b The Faraday Institution, Harwell Science and Innovation Campus, Didcot, OX11 0RA, United Kingdom

**Keywords:** Ultrasonic testing, Lithium-ion battery manufacturing, Electrode calendering, Coating, Production line parameterisation

## Abstract

Inline, non – destructive diagnostics are essential for controlling electrode quality in roll – to – roll battery manufacturing to minimise the cost and wasting valuable minerals. Ultrasonic pulse – echo is sensitive to thickness, density, and porosity, but open, process – aware datasets especially at electrode level remain limited and scarce. This data article disseminates open – access ultrasonic frequency – domain datasets, aligned manufacturing process metadata and machine – learning outputs, acquired during coating and calendering of lithium-ion battery electrodes. Unlike prior studies that reported time – domain analyses, this work releases the first curated frequency – domain ultrasonic dataset ath the electrode level. The data repository includes FFT frequencies and magnitudes, with mass, thickness, density, and full design of experiment factors. These data enable inspection of manufacturing – induced microstructural change without destruction and support benchmarking of signal-processing pipelines as well as development of inline, physics-informed quality control models for battery – electrode manufacturing.

Specifications Table

**Table:** 

Subject	Engineering & Materials science
Specific subject area	Ultrasonic testing and machine learning for parameterising coating and calendering of lithium-ion electrodes
Type of data	Processed frequency domain ultrasonic signals; Json files (frequency domain features & process factors); Python scripts for ML modelling
Data collection	Data has been collected by using an EPOCH-650 (10 MHz) under a load-controlled contact rig (cathodes used a 5 mm delay line). Acquisition parameters were held constant, and instrument control/logging were handled via a custom Python script. Thickness and mass were measured around each scan to compute density and align physical changes with the dominating frequency resonance.
Data source location	Warwick Manufacturing Group (WMG), University of Warwick, Coventry CV4 7AL, United Kingdom
Data accessibility	Repository name: Frequency-Domain Ultrasonic Signal Dataset for Battery Electrode Thickness Prediction Data identification number: 10.17632/c62yn37d9h.3 Direct URL to data: https://data.mendeley.com/datasets/c62yn37d9h/4 Use the DOI or URL link to access the repository which will direct you to the folders for Anode and Cathode datasets. Start with read me files for file structure, variables, and units. Cite the dataset DOI in any reuse. Each dataset folder contains a top – level Readme/Description file describing Phyton libraries, and versions, ML workflows, file structure, variable definitions, units, and citation guidance
Related research article	Guk, E.; Faraji Niri, M.; Farhadi Tolie, H.; Capener, M.; Bellchambers, P.; Marco, J. Investigation of calendaring parameters on the microstructure of graphite anodes by ultrasonic testing. *Journal of Power Sources* 614 (2024) 235,063. https://doi.org/10.1016/J.JPOWSOUR.2024.235063

## 1 Value of the Data

• **Importance**
  We present the first open access ultrasonic testing (UT) frequency – domain dataset at the electrode level, encompassing both anode and cathode samples scanned before and after proposed manufacturing process. The measurements were performed using an internally developed high-precision ultrasonic rig, designed to ensure a consistent and controlled testing environment. The dataset captures the influence of key manufacturing parameters on electrode microstructure, as reflected in the frequency-domain characteristics of the acquired ultrasonic signals.
  Structured according to *Data in Brief* best practices, the package includes a clear folder schema, parameter dictionaries, and links to related publications/data to enhance reusability and reproducibility.
  Finally, the dataset supports broader research applications, including cross-material meta-analyses, UT sensor selection and operating window optimisation, and acoustic non-destructive testing method development for porous composites. It is also suitable for educational use in physics and materials science curricula.
  While the underlying ultrasonic measurement originates from experiments reported in the related articles, that study focused on time – domain reflection of application specific analysis of anode electrode. In contrast, the present dataset provides a harmonised, frequency – domain dataset with structured metadata and more importantly scalable and reusable formats, which were not previously published or deposited elsewhere. This distinction ensures that the dataset serves as a canonical resource for spectral analysis, benchmarking, and ML development, rather than a repetition of prior results.
• **Target Audience**
  Researchers in battery manufacturing diagnostics, material characterisation, and ML practitioners building surrogate models for inline quality assurance and digitalisation of manufacturing process.
  Industrial engineers working on gigafactory quality assurance and digital twin development, and educators teaching UT/signal processing especially for practical session. Practitioners can derive roll-gap tolerance thresholds, train inline thickness estimators, and calibrate acoustic modules using descriptors validated in electrode studies.
• **Future Use**
  The dataset enables parameterisation of coating and calendering effects through frequency-domain acoustic features, providing spectral representations that capture process-induced changes in electrode microstructure. Observable trends – such as frequency shifts, bandwidth variations, and attenuation patterns associated with different coating weights and roll gaps – can be reproduced or extended using this dataset. These frequency-domain insights support the development of AI models both machine – and deep – learning for compaction, porosity, and mechanical uniformity monitoring without the need for destructive analysis.
  To support reproducible machine-learning (ML) research, the dataset provides frequency-domain signals derived from Fast Fourier Transform (FFT) – capturing key spectral peaks, bandwidths, and energy distributions linked to coating and calendering parameters. The dataset further contains the exact process parameter settings set to produce the electrodes, enabling researchers to use them as additional features for ML modelling inputs. Moreover, ML-ready assets, such as CatBoost baselines, Swin-Transformer models, and a conditional GAN for thickness-aware data augmentation are provided as an initial foundation. Ablation studies are provided to benchmark classical versus deep-learning approaches and to facilitate transfer learning across electrode types and production conditions.

## 2 Background

Ultrasonic testing (UT) provides a non-destructive means of monitoring microstructural evolution – including thickness variation, densification, and porosity reduction – during electrode coating and calendering [1,2]. In previous studies, time-domain analyses of graphite anodes and Ni-rich NMC-622 cathodes revealed measurable effects of roll gap, line speed, and coating parameters on the backwall echo (time of flight and amplitude). Building on that foundation, the present work introduces a harmonised frequency – domain dataset, offering spectral representations that capture process-dependent shifts in frequency, bandwidth, and attenuation. By coupling these frequency – domain features with detailed process parameters, the dataset enables quantitative parameterisation of coating and calendering conditions and supports inline quality assurance without destructive sectioning.

In line with *Data in Brief* practice, the emphasis is on reusability and benchmarking: researchers in both academia and industry can use this dataset to compare feature – extraction pipelines, derive physics – informed correlations between spectral response and microstructural compaction, and train surrogate models for process optimisation and control. This dataset complements our related research on anode and cathode ultrasonic characterisation and model development [3] by providing the complete signal sets, derived frequency – domain features, and associated process parameters for independent analysis and model refinement.

## 3 Data Description

The dataset comprises ultrasonic frequency-domain data acquired from graphite anode and Ni-rich NMC-622 cathode electrodes manufactured under controlled coating and calendering conditions. Each electrode was scanned at three marked locations before and after calendering to enable direct comparison of identical regions. The associated design of experiments (DoE) covers variations in roll gap, line speed, and coat weight for cathodes, and roll gap and calendering speed for anodes. Raw ultrasonic signals were transformed into frequency-domain representations using Fast Fourier Transform (FFT), producing spectral features that characterise process-induced microstructural changes.. The ultrasonic signals were collected using a high-precision in-house testing setup and processed to generate both time-domain waveforms and frequency – domain representations, i.e. Fast Fourier Transform. These paired acoustic and process data form a unified, reusable resource for analysing how manufacturing parameters influence electrode microstructure.

**Fig. 1:**
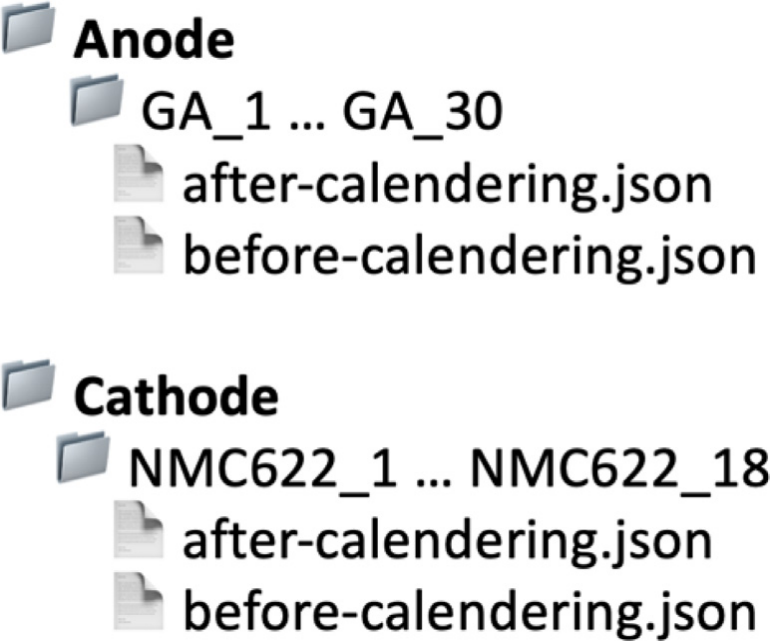
Directory structure of anode and cathode data.

Fig. 1 illustrates the dataset directory structure, organised for immediate reuse. Two top-level folders, Anode and Cathode, contain sample-specific subfolders (GA_1 to GA_30 for anode; NMC622_1 to NMC622_18 for cathode), each holding the after – calendering.json and before – calendering.json files with the corresponding frequency domain ultrasonic data along with the process parameters and material properties including thickness and density. In addition, another top-level folder, Machine Learning, provides python scripts to train and test ML models to predict the thickness of electrodes using the provided frequency domain signals. A lightweight Python script is also provided to facilitate rapid inspection of the ultrasonic data. The script loads the JSON files for any selected sample, extracts FFT frequency and magnitude arrays, and generates a comparative stem plot visualising the spectral response before and after calendering. Metadata such as the sample ID and calendering state are automatically incorporated into the plot, ensuring consistent and interpretable visualisation. This utility enables users to validate the data structure, inspect signal integrity, and quickly assess the influence of the calendering process on ultrasonic response characteristics.

Each dataset is organised so that every sample is stored in its own folder as two JSON files – before-calendering.json and after – calendering.json – containing both metadata and ultrasonic frequency – domain features.

• For cathodes, each JSON file provides metadata fields such as Sample_ID, Web Speed, Coat Weight, Roll Gap, Thickness, Density, and the Calendering_State. The ultrasonic content includes the full FFT frequency array (typically spanning ∼1–15 MHz) and the corresponding FFT magnitude vector, representing the spectral response of the electrode. All physical variables stored in the metadata explicitly include unit (e.g., thickness in µs, density in g.cm^-3^ roll gap in µm, and web speed in m.min^-1^), as documented in the accompanying Readme/Description files to ensure unambiguous reuse.
• For anodes, each JSON file follows the same structure, with metadata including Calendering Speed, Roll Gap, Thickness, and Density, along with FFT frequency and FFT magnitude arrays derived from the calibrated ultrasonic measurements.
• Both datasets expose the complete FFT-frequency axis directly within the JSON files, enabling straightforward mapping between spectral amplitude and physical frequency.

**Fig. 2:**
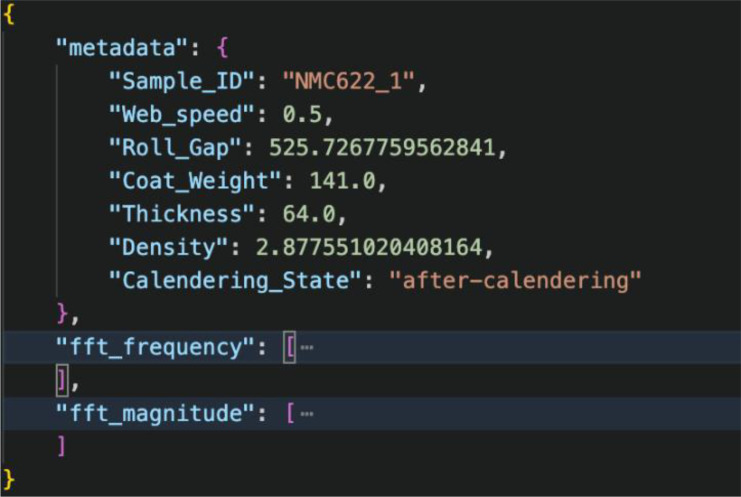
Sample JSON structure for a cathode electrode (NMC622_1), showing the stored metadata together with the FFT frequency array and corresponding magnitude spectrum derived from the ultrasonic measurement.

In this updated release, the frequency-domain information within each JSON file serves as a complete feature set – containing the FFT vectors, spectral peaks, bandwidth characteristics, and energy – based descriptors computed from the magnitude spectra. These frequency – domain representations complement the ultrasonic signal structure and enable users to assess coating quality and calendering effects through spectral analysis (Fig. 2).

The repository includes machine – learning (CatBoost) and link to deep-learning models (multi – scale CNN with Swin Transformer modules) tailored for both anode and cathode datasets, along with a conditional generative adversarial network (cGAN) pipeline for synthetic signal generation. Comprehensive documentation and example scripts are provided to ensure full reproducibility and to support model comparison, feature extraction, and transfer learning across materials and production conditions.

## 4 Experimental Design, Materials and Methods

A structured design of experiments (DoEs) [1,2] was used to generate ultrasonic data from graphite anode and Ni-rich NMC – 622 cathode electrodes produced under controlled coating and calendering conditions. The DoE systematically varied coating and calendaring parameters to capture their combined influence on ultrasonic response. Each electrode was scanned at three marked locations before and after calendering, enabling direct comparison of identical positions and consistent evaluation of process effects.

Electrode samples were prepared on metallic current collectors using standard slurry – based coating and calendaring procedures. Manufacturing and testing were carried out at the WMG Battery Scale-Up facility. All samples were cut into circular discs compatible with a custom – built ultrasonic test rig that ensured stable coupling and repeatable measurement geometry.

Ultrasonic testing employed a pulse – echo method with a broadband transducer suitable for electrode – level characterisation. Data acquisition was fully automated through custom scripts linking measurement parameters to corresponding process conditions.

Each raw waveform was averaged from multiple readings per location and processed using Python code to obtain both time-domain signals and frequency – domain representations. Derived frequency – domain representations and spectral descriptors were compiled into structured JSON files aligned with process metadata, without reproducing analytical results reported in the associated research article.

The final dataset was curated following *Data in Brief* standards, with harmonised variable names, detailed metadata, and accompanying Readme/Description files to ensure clarity, reproducibility, and ease of reuse.

## Limitations

The dataset was collected using a single-frequency (10 MHz) pulse – echo configuration with contact and delay – line arrangements for the anode and cathode samples, respectively. This setup constrains absolute velocity estimation in very thin multilayer structures and limits spatial coverage to three marked positions per electrode. Nevertheless, the measurements were conducted under carefully controlled coupling and acquisition conditions, with automated logging and re – acquisition of the same positions before and after calendering, ensuring signal consistency and process-traceable comparisons.

A key enhancement in this dataset is the inclusion of a comprehensive frequency-domain representation of all ultrasonic signals, allowing researchers to analyse spectral behaviour – such as shifts in dominant frequency, bandwidth, and attenuation patterns – linked to coating and calendering parameters. This frequency – domain layer significantly increases the dataset’s analytical value and reusability by enabling spectral comparison across materials and process conditions, and by supporting transferable, physics-informed modelling that was not feasible using time-domain data alone.. Future work may extend the dataset through multi – frequency and spatially denser acquisitions to further broaden its applicability for model development and process optimisation.

## Ethics Statement

The proposed data does not involve any human subjects, animal experiments, or data collected from social media platforms. The authors confirm that this work meets the ethical requirements of the journal.

## CRediT Author Statement

**Erdogan Guk:** Writing – review & editing, Writing – original draft, Conceptualisation, Visualization, Validation, Methodology, Formal analysis, Data curation. **Hamidreza Farhadi Tolie:** Writing – review & editing, Formal analysis, Data curation. **Mona Faraji Niri:** Writing – review & editing, data curation. **James Marco:** Writing – review & editing, Writing – original draft, Validation, Supervision, Resources, Project governance.

## Data Availability

Mendeley DataFrequency-Domain Ultrasonic Signal Dataset for Battery Electrode Thickness Prediction (Original data)
